# Accuracy and applicability of dual-energy computed tomography in quantifying vertebral bone marrow adipose tissue compared with magnetic resonance imaging

**DOI:** 10.1186/s13244-022-01326-0

**Published:** 2022-11-26

**Authors:** Zhenghua Liu, Dageng Huang, Yuting Zhang, Rong Chang, Xiaoyue Zhang, Yonghong Jiang, Xiaowen Ma

**Affiliations:** 1 Department of Radiology, Honghui Hospital Affiliated Xi’an Jiaotong University, No. 555, Youyi East Road, Xi’an, 710054 China; 2 Department of Spinal Surgery, Honghui Hospital Affiliated Xi’an Jiaotong University, Xi’an, China; 3 Siemens Healthcare Limited, Xi’an, China

**Keywords:** Dual-energy computed tomography, Marrow adipose tissue, Osteoporosis, Magnetic resonance imaging, Bone density

## Abstract

**Objectives:**

To evaluate the accuracy of dual-energy computed tomography (DECT) in quantifying bone marrow adipose tissue (BMAT) and its applicability in the study of osteoporosis (OP).

**Methods:**

A total of 83 patients with low back pain (59.77 ± 7.46 years, 30 males) were enrolled. All patients underwent lumbar DECT and magnetic resonance imaging (MRI) scanning within 48 h, and the vertebral fat fraction (FF) was quantitatively measured, recorded as DECT-FF and MRI-FF. A standard quantitative computed tomography (QCT) phantom was positioned under the waist during DECT procedure to realize the quantization of bone mineral density (BMD). The intraclass correlation coefficient (ICC) and Bland–Altman method was used to evaluate the agreement between DECT-FF and MRI-FF. The Pearson test was used to study the correlation between DECT-FF, MRI-FF, and BMD. With BMD as a gold standard, the diagnostic efficacy of DECT-FF and MRI-FF in different OP degrees was compared by receiver operating characteristic (ROC) curve and DeLong test.

**Results:**

The values of DECT-FF and MRI-FF agreed well (ICC = 0.918). DECT-FF and MRI-FF correlated with BMD, with r values of −0.660 and −0.669, respectively (*p* < 0.05). In the diagnosis of OP and osteopenia, the areas under curve (AUC) of DECT-FF was, respectively, 0.791 and 0.710, and that of MRI-FF was 0.807 and 0.708, and there was no significant difference between AUCs of two FF values (with Z values of 0.503 and 0.066, all *p* > 0.05).

**Conclusion:**

DECT can accurately quantify the BMAT of vertebrae and has the same applicability as MRI in the study of OP.

## Key points

- Quantification of BMAT valuable information for the clinical diagnosis and treatment of OP.
- DECT can accurately quantify the BMAT of vertebrae.
- DECT has the same applicability as MRI in the study of OP.

## Introduction

Osteoporosis (OP) is a complex multifactorial disease, mainly manifested as a decrease in bone density and bone strength, and its diagnosis and quantitative assessment mainly rely on bone density assay [1]. Recent studies have shown that bone marrow adipose tissue (BMAT) plays an important influence in the mechanism of OP, and its endocrine effect and impact on bone structural strength is worth noting [2, 3]. BMAT may be a biomarker of osteoporosis (OP) [4, 5], and greater BMAT content is associated with greater losses of the bone density and bone compressive strength [6]. Therefore, accurate quantification and evaluation of BMAT can provide valuable information for clinical diagnosis and treatment of OP [5, 7].

Due to the existence of BMAT, in some studies on vertebral bone density assay and fracture risk assessment, Magnetic resonance imaging (MRI) or dual-energy computed tomography (DECT) is used to quantify BMAT to correct the bone density error caused by it [8, 9]. MRI is currently the best method for fat quantification, including proton magnetic resonance spectroscopy (1H-MRS), water fat imaging, such as iterative decomposition of water and fat with echo asymmetry and least-squares estimation (IDEAL-IQ). As a mature technology, it is widely used in studies on fat quantification of soft tissue, bone, and tumor [10–12].

In contrast, DECT is a rapidly developing new technology, which is based on high and low-energy material separation technology that can quantify specific substances, such as calcium and fat [13]. Recent studies have found that DECT has shown potential in fat quantification. Cao's research has suggested that multi-parameter imaging of DECT can accurately quantify the fat content of liver to evaluate the severity of liver fat deposition [14]. In Baillargeon's study, they quantified the fat infiltration of skeletal muscle by DECT and found that the DECT muscle fat fraction (FF) showed excellent correlation with clinically accepted standards [15]. However, few studies have been reported on DECT to quantify BMAT, as MRI is the most recognized and accurate method.

In previous studies, we quantified the BMAT and calcium density of vertebrae by DECT, which provides valuable information for the clinical diagnosis and treatment of OP [16]. We hope to further evaluate the accuracy of DECT in quantitative BMAT and whether it is suitable for the quantitative evaluation of OP compared to MRI. This work aimed to study the agreement of DECT and MRI in quantification of vertebral BMAT and to evaluate the applicability of DECT in the study of OP.

## Methodology

### Study design

**Fig. 1:**
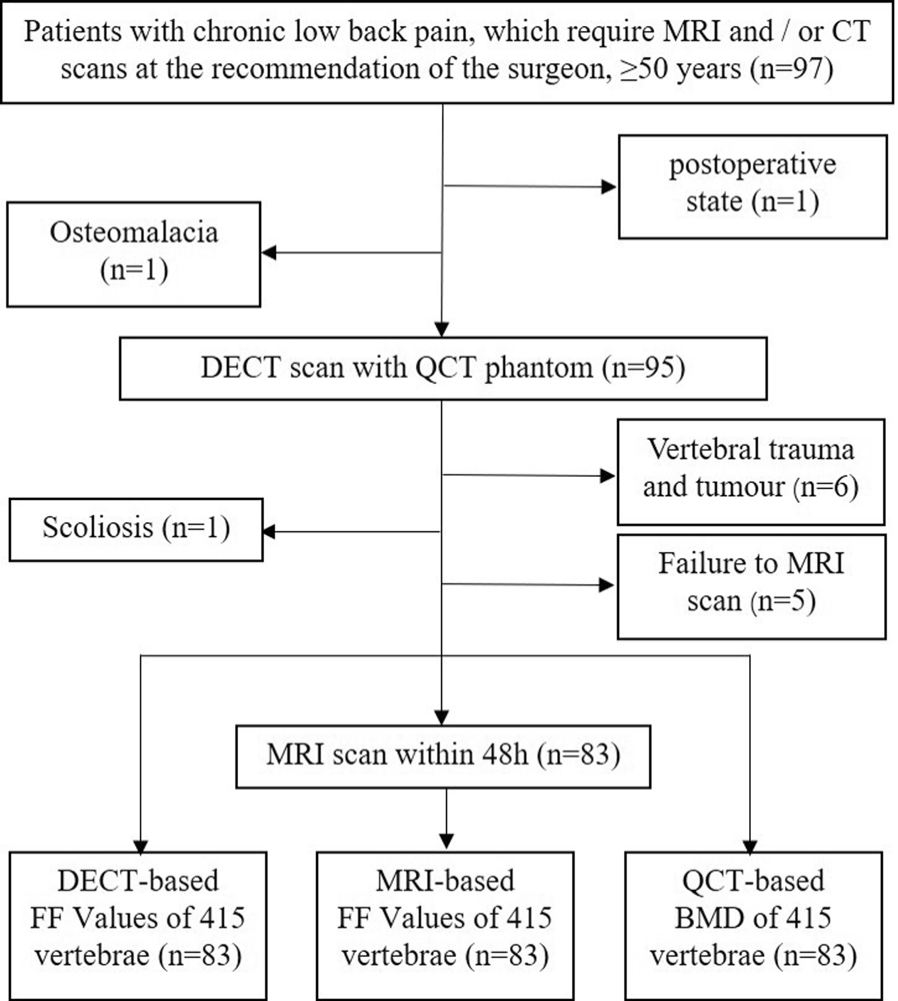
The flowchart of the study. *DECT* Dual-energy computed tomography, *QCT* quantitative computed tomography, *MRI* magnetic resonance imaging, *FF* fat fraction, *BMD* bone mineral density

The present study was conducted following the Declaration of Helsinki (as revised in 2013) and approved by the Ethics Committee of our hospital (IRB No. 201902068). This is a secondary analysis of a prospective study, and some patients with chronic low back pain underwent MRI and/or CT scans at the recommendation of surgeons. With full communication and informed consent, some patients who underwent lumbar MRI and CT scanning were initially included in the study. Inclusion criteria were as follows: ① age should be ≥ 50 years; ② lumbar DECT and MRI scan within 48 h. Exclusion criteria included the following points: ① scoliosis; ② localized osteosclerosis in vertebral cancellous bone; ③ vertebral trauma and tumor; ④ postoperative state of lumbar vertebra. The flowchart displaying patient inclusion of this study is shown in Fig. 1.

A total of 83 patients with chronic low back pain from April to November in 2021 were enrolled, including 30 males and 53 females. By placing a standard QCT corrected phantom (QCT Pro v5.0; Mindways, Tex) under the waist during DECT procedure, we obtained DECT parameters and QCT-based bone mineral density (BMD) concurrently. Thus, no additional radiation exposure was entailed.

### DECT scanning and vertebral BMAT quantification

DECT examinations relied on a second-generation 128-section dual-source unit operating in dual-energy mode (Somatom Definition Flash; Siemens Healthineers, Erlangen, Germany). Settings of both x-ray tubes were constant (tube A: 80 kV, 250 mAs; tube B: 140 kV with Sn filter, 97 mAs), pitch of 0.6, collimation width 32 × 0.6 mm, rotation time 500 ms/r, field of view (FOV) 500 mm × 500 mm. The scanning range extended from the 12^th^ thoracic vertebra to the 1^st^ sacral vertebra. Images were reconstructed using a kernel of I30f, 1-mm section thickness, and 0.75-mm increment. All radiation doses received by patients were recorded upon completion.

**Fig. 2:**
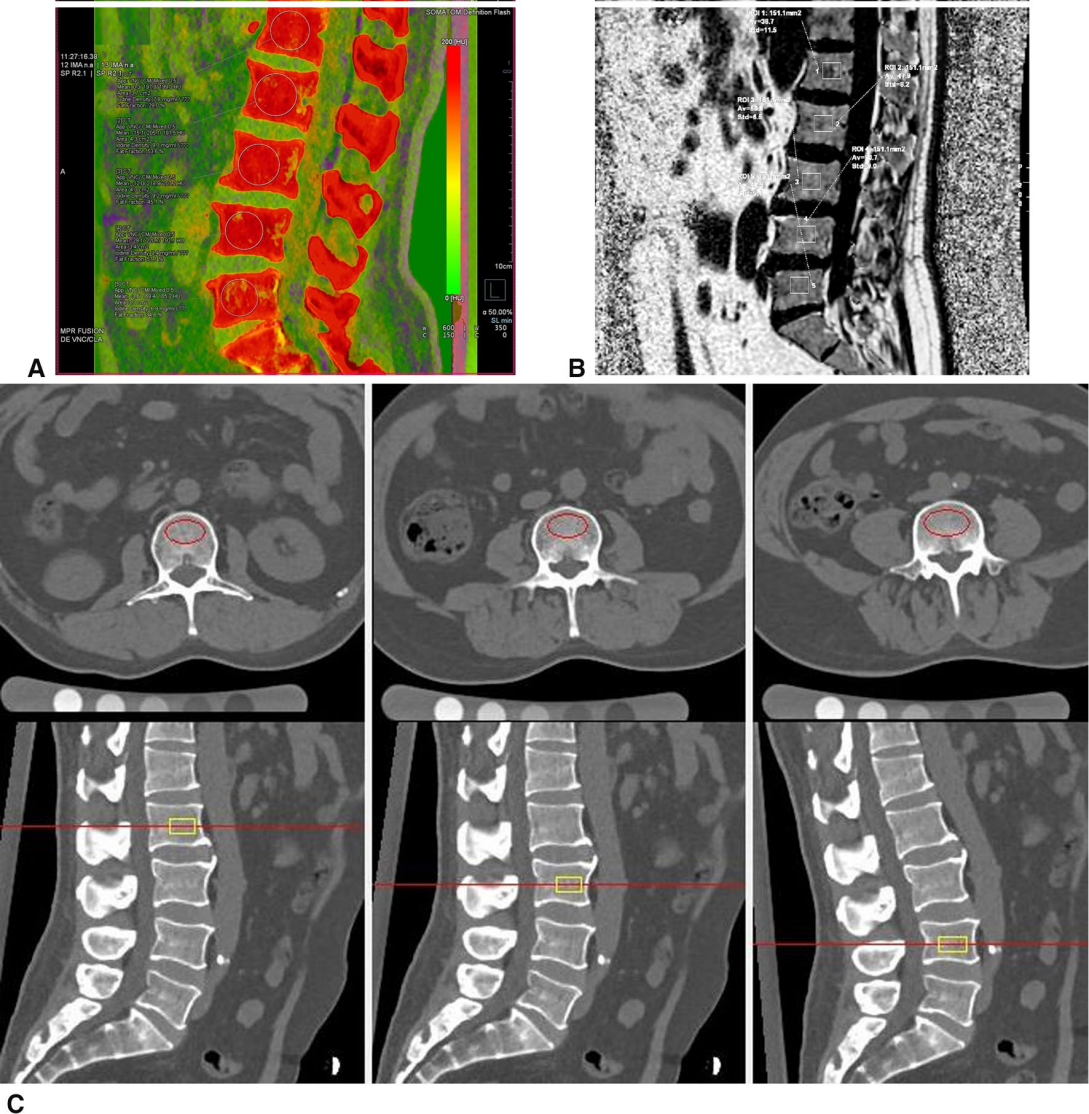
Measurement of DECT-FF, MRI-FF, and BMD: **A**, **B** DECT-FF and MRI-FF derived from corresponding region of interest (ROI) in a standard median sagittal plane, respectively, and the ROI was delineated in 2/3 of the anterior vertebral body, avoiding the bone cortex, vertebral vein sulcus, and surrounding osteosclerosis; **C** BMD determined by ROI automatically drawn with QCT analytics system

The quantitative measurement of the vertebral BMAT was carried out based on the liver virtual non-contrast function module of the dual-energy analytic software (Syngo. via VB10; Siemens Healthcare, Erlangen, Germany). According to the parameter settings in bone marrow analysis, the default value of soft tissue was modified to 55 HU and 51 HU, fat value to − 110 HU and − 87 HU, and iodine slope to 1.71 [16]. The FF values of 1st to 5th lumbar vertebrae were measured in the median sagittal view, and the mean FF value of each patient was recorded as DECT-FF (Fig. 2a).

### MRI scanning and vertebral BMAT quantification

MRI examinations relied on a 3.0 T superconducting MR scanner (Discovery 750, GE Healthcare, Milwaukee, WI, USA), with standard human body coil and sagittal scanning. Prior to IDEAL-IQ, T1 weighted image (T1WI) (repetition time [TR]/ time to echo [TE] = 400/13 ms), T2WI (TR/TE = 2500/102 ms), FOV 36 cm × 36 cm, matrix of 224 × 192, pixel size 1.6 mm × 1.9 mm, slice thickness of 3 mm, intersection gap of 0.4, number of excitations (NEX) of 1; IDEAL-IQ: TR of 7.4 ms, minimum TE of 1.3 ms, maximum TE of 5.3 ms, flip angle of 4°, echo train length of 5, bandwidth of 111.1 kHz, and other settings were the same as above.

Four group images were obtained once the IDEAL-IQ sequence was scanned: pure water image, pure fat image, fat fraction image, and R2* relaxation rate image. The FF value was measured on the viewer module of ADW4.7 workstation. Select the fat fraction image, draw a rectangular ROI on the first 2 / 3 of the vertebral body in the median sagittal diagram, then the FF values of 1st to 5th lumbar vertebrae were measured successively at one slice (Fig. 2b), the mean FF value of each patient was recorded as MRI-FF.

### QCT-based BMD measurement and grouping

DECT equipment and QCT analytics (QCT Pro v5.0; Mindways, Tex) were calibrated in advance using a quality control phantom. The scan data of mixed ratio (0.5) were imported to the QCT software application, and the BMD values of 1st to 5th lumbar vertebrae were measured by drawing a region of interest automatically (Fig. 2c). Then, the mean BMD value of 5 vertebrae was taken as the patient's BMD. According to BMD, all patients were divided into normal group (BMD > 120 mg/cm^3^), osteopenia group (120 mg/cm^3^ ≥ BMD > 80 mg/cm^3^) and OP group (BMD ≤ 80 mg/cm^3^) [17].

### Statistical analysis

All vertebral measurements were performed independently by 2 experienced radiologists, who had been engaged in musculoskeletal imaging research for more than 8 years, and take the mean value of 2 measurers as the final value. The consistency analyses for the measurements of 2 measurers were performed by intraclass correlation coefficient (ICC).

Statistical analysis was performed using Medcalc (version 19.0 MedCalc Software bvba, Belgium). Variables were tested for normality of distribution by Shapiro–Wilk test, and expressed as means ± SDs. The difference between normal, osteopenia and OP groups and between 1st and 5th vertebra were tested by *one-way ANOVA,* and the difference of FF values between adjacent vertebrae was tested by *independent sample t test*. The consistency between DECT-FF and MRI-FF was analyzed by *ICC* and *Bland–Altman method* (ICC of < 0.4 means poor consistency, ICC of 0.4–0.75 means general consistency, ICC of > 0.75 means good consistency); the *Pearson* correlation analysis was used to analyze the correlation between DECT-FF, MRI-FF, and BMD (|r| of < 0.5 means low linear relationship, |r| of 0.5–0.8 means a significant linear relationship, and |r| of > 0.8 means a highly linear relationship). Taking BMD as the gold standard, DECT-FF and MRI-FF diagnostic efficacy in different OP degrees were evaluated by receiver operating characteristic (ROC) curve, and the area under the curve (AUC) of DECT-FF and MRI-FF were compared by the *DeLong* test (AUC of 0.5–0.7 means low diagnostic value, AUC of 0.7–0.9 means moderate diagnostic value, and AUC of > 0.9 means high diagnostic value). A *p* value of < 0.05 was statistically significant.

## Results

A total of 83 patients were enrolled in this study. Of them, 30 were males and 53 were females, aged from 50 to 88 years, with an average age of 59.77 ± 7.46 years. The volume CT dose index was 9.53 mGy, the average dose-length product was 332.36 ± 40.26 mGy·cm, and the mean radiation dose was 4.61 ± 0.59 mSv. The quantitative parameters of all vertebrae measured by 2 measurers were in good agreement, including FF values based on DECT and MRI and BMD based on QCT (ICC = 0.905, 0.917, 0.938, respectively).

**Fig. 3:**
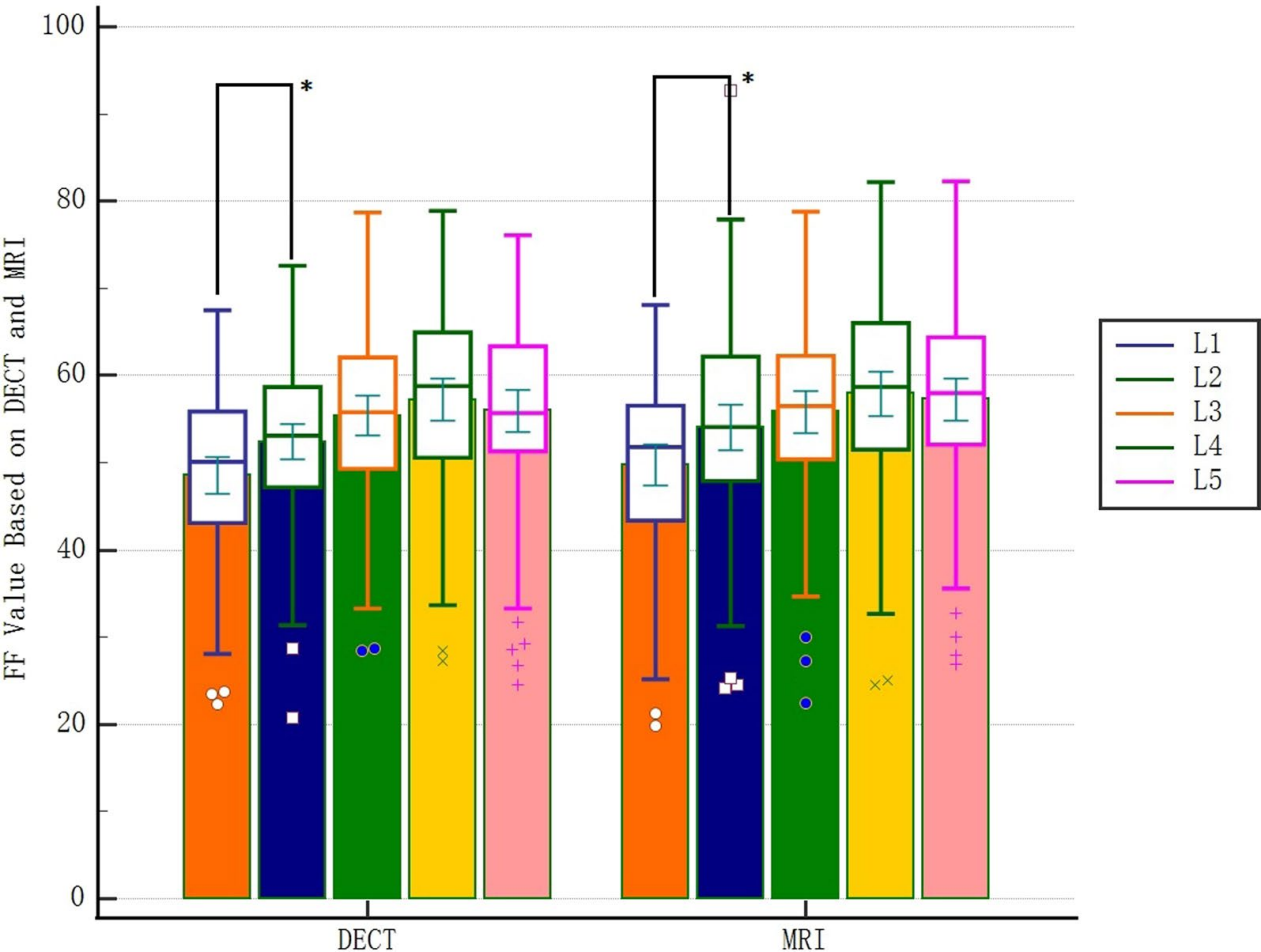
The FF values of 1st to 5th vertebrae based on DECT and MRI. *p < 0.05 DECT: Dual-energy computed tomography, *MRI* magnetic resonance imaging, *FF* fat fraction

There was no significant difference in BMI between normal, osteopenia and OP groups, but there was significant difference in BMD, DECT-FF and MRI-FF (Table 1). The column diagram showed that FF values of 1^st^ to 5^th^ vertebra tended to increase gradually, including DECT-FF and MRI-FF; One-way ANOVA showed that there was no significant difference in FF values between 1st and 5th vertebra; Independent sample t test showed that there was a statistical difference in FF values between 1st and 2nd vertebra, which did not exist between 2nd and 3rd, 3rd and 4th, 4th and 5th vertebra (Fig. 3).

**Table 1:** General conditions and measurements of patients in different OP groups (n = 83)

	Normal (22)	Osteopenia (28)	OP (33)	Total (83)	F	*p*
Age (year)	56.45 ± 6.12	59.36 ± 6.44	62.33 ± 8.27	59.77 ± 7.46	4.527	0.014
BMI (kg/m^2^)	25.76 ± 2.61	24.14 ± 3.25	24.8 ± 2.96	24.83 ± 3.01	1.838	0.166
BMD (mg/cm^3^)	134.59 ± 20.32	94.63 ± 8.43	58.44 ± 15.79	90.83 ± 34.17	166.201	< 0.001
DECT-FF (%)	45.87 ± 10.71	53.73 ± 6.9	59.36 ± 6.12	53.89 ± 9.43	19.658	< 0.001
MRI-FF (%)	46.28 ± 12.34	54.41 ± 7.24	61.22 ± 6.67	54.96 ± 10.47	19.611	< 0.001

*OP* osteoporosis, *BMI* body mass index, *BMD* bone mineral density, *DECT-FF* fat fraction based on Dual-energy computed tomography, *MRI-FF* fat fraction based on magnetic resonance imaging

### Consistency analysis

**Fig. 4:**
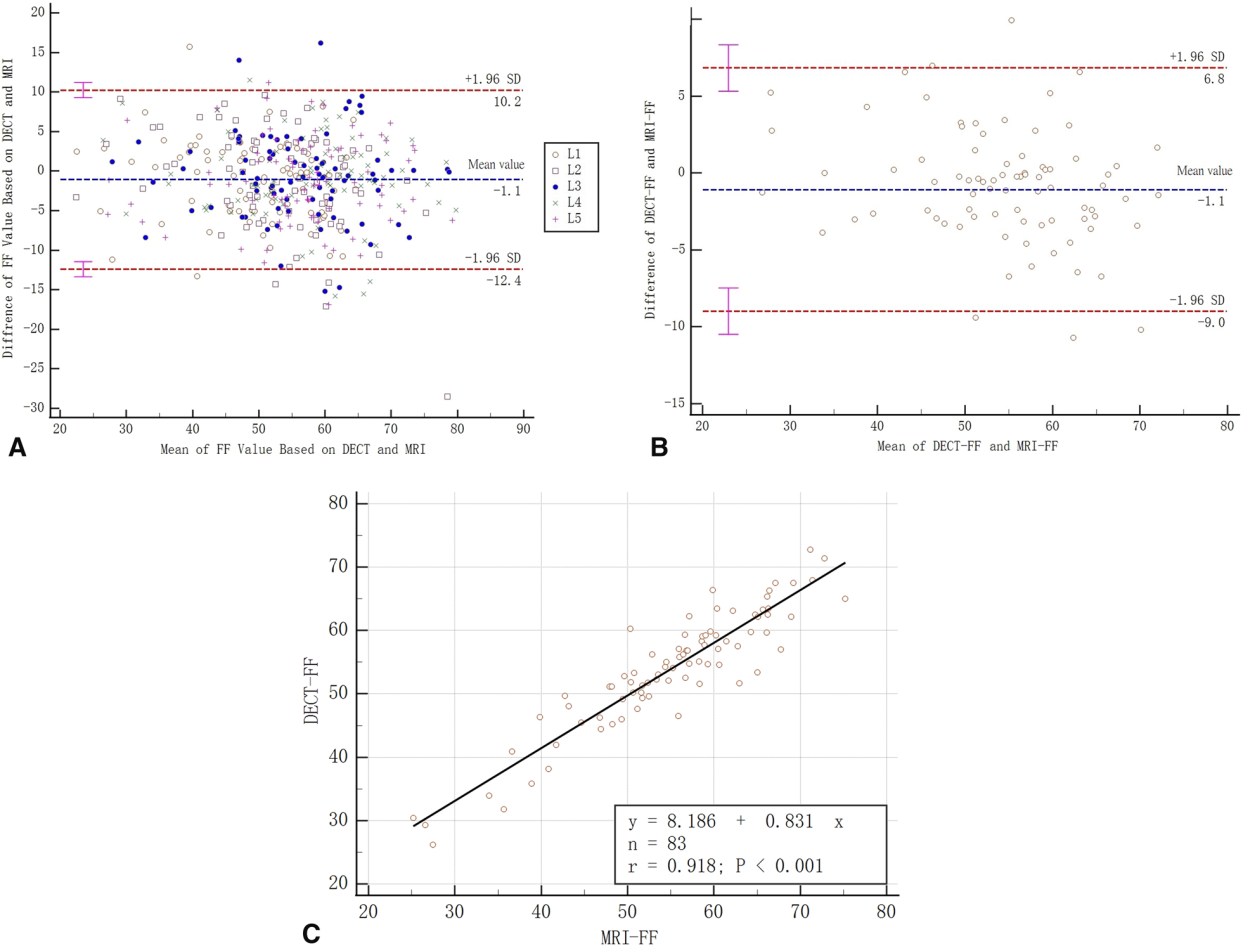
Bland–Altman plots for the agreement between EDCT and MRI. **A** The agreement between the FF values of all 415 vertebrae based on EDCT and MRI. **B** Bland–Altman plots for the agreement between the FF values of all 83 parents based on EDCT and MRI. **C** DECT-FF and MRI-FF were linearly correlated, with a slope of 0.831 and an intercept of 8.186. DECT: Dual-energy computed tomography, MRI: magnetic resonance imaging, FF: fat fraction

The FF value of all 415 vertebrae measured based on two methods were in good agreement (ICC = 0.865). In comparison, the measured value of DECT-FF and MRI-FF of each patient had higher consistency (ICC = 0.918), since they were the average of 5 lumbar vertebrae. As all mean differences tend to be zero and most of the differences lay between ± 1.96 SD, Bland–Altman plots indicate high agreement between both quantization method (Fig. 4AB). Linear regression analysis showed that DECT-FF and MRI-FF were linearly correlated (r = 0.918, *p* < 0.001), with a slope of 0.831 and an intercept of 8.186 (Fig. 4C).

### Correlation analyses

**Fig. 5:**
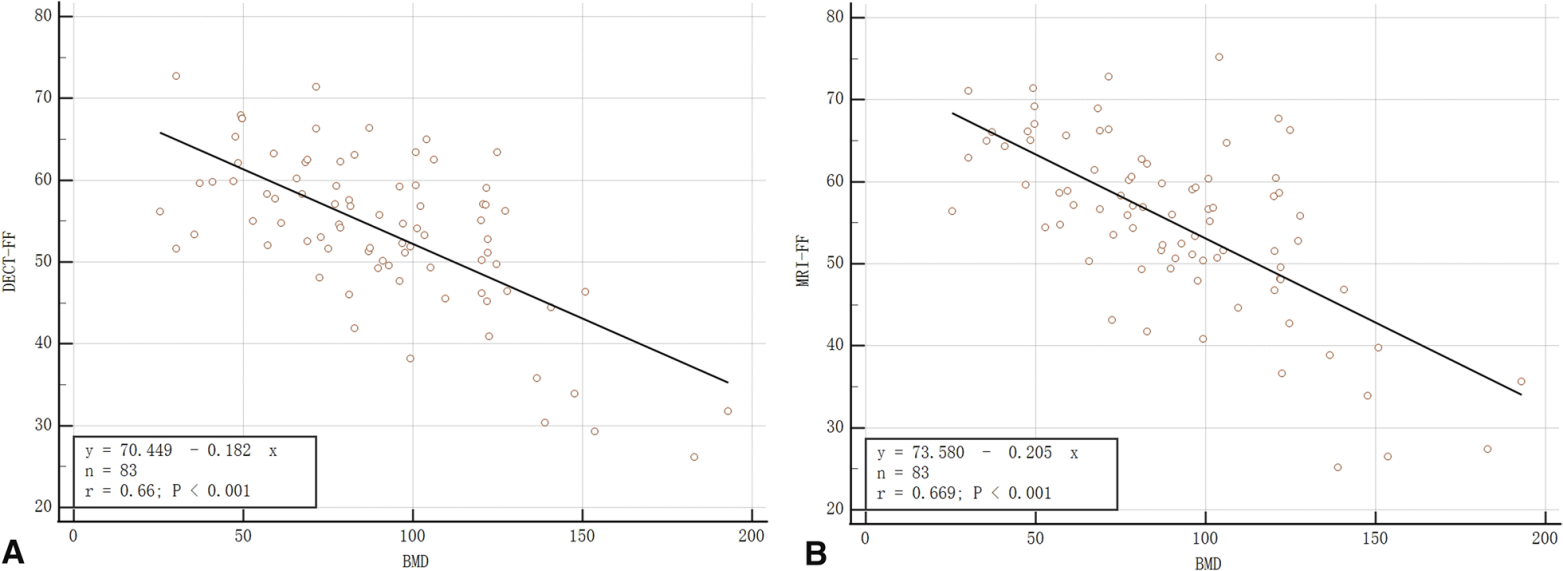
Correlation between DECT-FF, MRI-FF, and BMD. **A**, **B** DECT-FF and MRI-FF were correlated with BMD, with R-values of −0.660 and −0.669, respectively. *DECT* Dual-energy computed tomography, *MRI* magnetic resonance imaging, *FF* fat fraction, *BMD* bone mineral density

Pearson's test showed a negative linear relationship between DECT-FF and MRI-FF and BMD. The correlation was significant, with r values of −0.660 and −0.669, respectively (both *p* < 0.001) (Fig. 5).

### Diagnostic accuracy

**Fig. 6:**
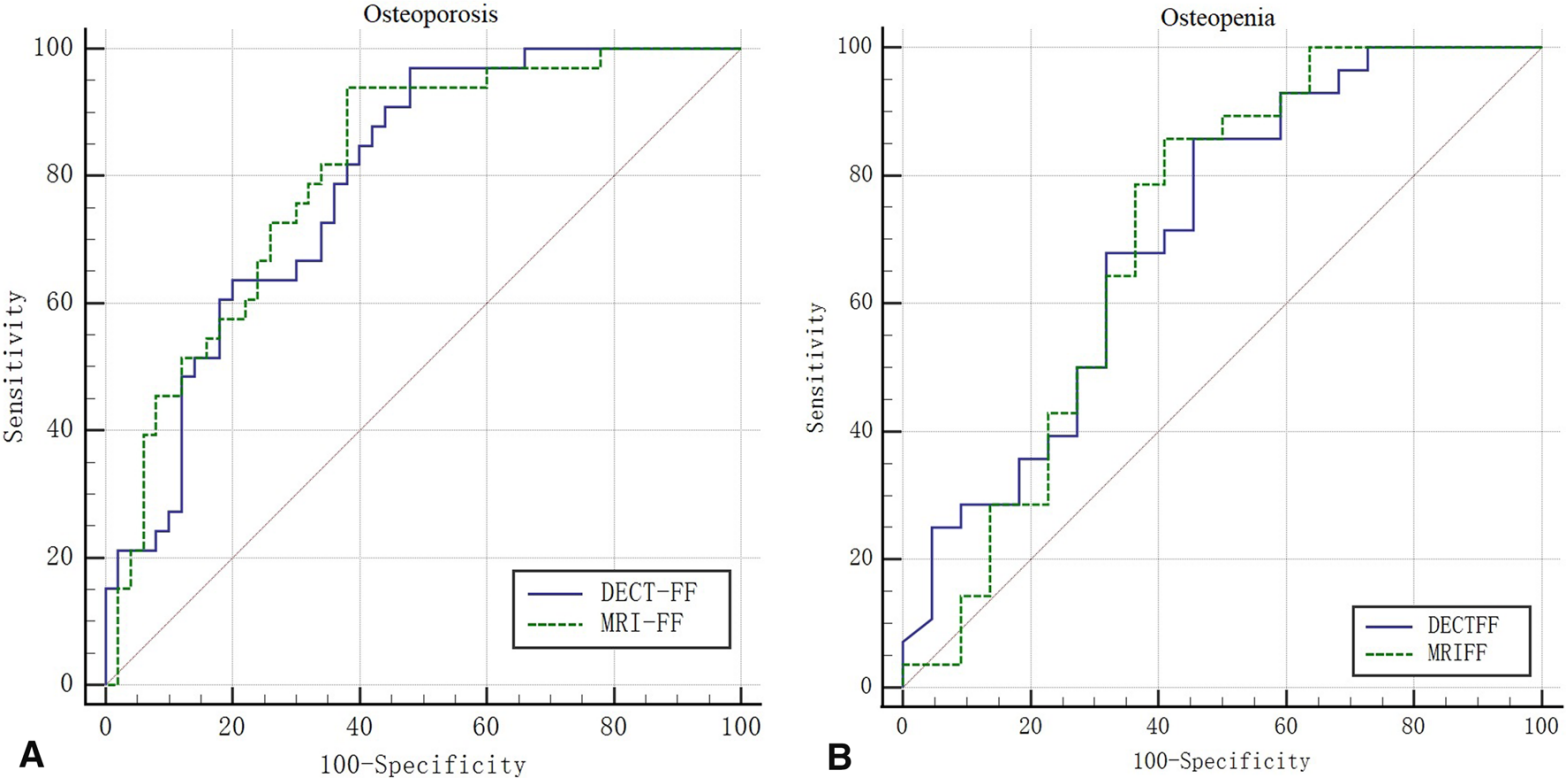
ROC curve of DECT-FF and MRI-FF in OP and Osteopenia diagnosis. **A** The AUC of DECT-FF and MRI-FF in the diagnosis of OP were 0.791 and 0.807, respectively; **B** The AUC of DECT-FF and MRI-FF in the diagnosis of Osteopenia were 0.710 and 0.708, respectively. DECT: Dual-energy computed tomography, MRI: magnetic resonance imaging, FF: fat fraction

The ROC curves of DECT-FF and MRI-FF in diagnosing OP and Osteopenia are presented in Fig. 6, and the threshold, sensitivity, specificity, and AUC are shown in Table 2. The AUCs of both DECT-FF and MRI-FF are not very high and they have moderate diagnostic value for OP and osteopenia. Also, from the table we can find there were no significant differences in AUC between DECT-FF and MRI-FF.

**Table 2:** Comparison of DECT-FF and MRI-FF in the evaluation of OP and osteopenia (n = 83)

		Threshold	Sensitivity	Specificity	AUC (95%*CI*)	*Z*	*p*
OP	DECT-FF	51.3	96.97	57.00	0.791 (0.688–0.872)	0.503	0.615
	MRI-FF	53.34	93.94	62.00	0.807 (0.706–0.886)		
Osteopenia	DECT-FF	46.48	85.71	59.55	0.710 (0.565–0.830)	0.066	0.948
	MRI-FF	48.2	85.71	64.09	0.708 (0.562–0.828)		

*DECT-FF* fat fraction based on Dual-energy computed tomography, *MRI-FF* fat fraction based on magnetic resonance imaging, *OP* osteoporosis, *AUC* area under the curve

## Discussion

In this study, we tried to evaluate the accuracy and applicability of DECT in quantifying vertebral BMAT, and found that the vertebral FF values based on DECT and MRI were in good agreement, and they reflected the same efficiency in the relevant studies of OP, which enhanced our confidence in further OP research using DECT.

In bone marrow, osteoblasts and adipocytes originate from a common type of mesenchymal stem cell in the bone marrow, and many osteoporotic states, including aging, nutritional fluctuations, hormonal changes, and metabolic disorders, such as obesity and diabetes, are associated with the marrow fat transformation [18]. BMAT could not only lead to marrow fat transformation but could also inhibit osteoblast differentiation and proliferation, resulting in diminished bone formation and, potentially, loss of bone mass leading to osteoporosis [19, 20]. For these reasons, the quantification of BMAT is particularly important in OP related studies, and the imaging technology advancements allow the noninvasive quantification of BMAT [21–23].

DECT is a rapidly developing imaging method for quantitative measurement. In conjunction with the VNC technique, DECT can be used for quantitative analysis of specific substances, such as iron, iodine, and fat [24–27]. For the spine, the CT density of the bone marrow cavity reflects the average density of three tissues: trabecular bone, BMAT, and hematopoietic tissue, corresponding to calcium, fat, and soft tissue, respectively. We modified the parameters in the liver VNC configuration file preset by the equipment manufacturer to the characteristic slope value of calcium, and the reference values of soft tissue and fat are accordingly modified to quantify the calcium and fat in vertebral cancellous bone. In this study, the FF value of the 1st to 5th lumbar spine showed a gradual increasing trend, which was consistent with previous studies [28].

This study revealed highly consistent quantitative parameters of vertebral BMAT (DECT-FF and MRI-FF) obtained by DECT and MRI equipment, respectively, indicating that DECT-FF could be used as an accurate and reliable parameter in vertebral BMAT quantification. This is consistent with Bredella's study [22], but IDEAL-IQ was selected as the control instead of 1H-MRS in our study. IDEAL-IQ technology realizes accurate quantification of fat content through the multi-echo collection and has the advantages of short scanning time and simple operation, compared with 1H-MRS sequence [28–30]. In addition, we also compared the diagnostic efficacy of DECT-FF and MRI-FF in different OP grades, and found that they had similar correlation, threshold, sensitivity and specificity, and there was no significant difference in AUC between them. DECT has the potential to become an alternative to MRI in the quantification of vertebral BMAT.

It should be noted that QCT-based BMD, as the gold standard for OP evaluation and grouping in this study, was considered to need correction due to the content of BMAT [8, 28]. However, this does not affect the reliability of our study, in which it was used as a reference to evaluate the consistency of DECT and MRI in quantifying BMAT and evaluating OP. On the contrary, this study also indirectly indicated that BMAT was an unavoidable problem in the process of bone densitometry. FF value can indirectly reflect bone mineral loss with a significant linear relationship with BMD, but it is not suitable as an independent diagnostic indicator of OP and their AUCs are not very high. Compared to DEXA, the FF value we obtained is a volumetric and more accurate measure, independent of overlap. Quantification of BMAT with FF allows correction of QCT-BMD, as suggested by some studies [8, 28], and DECT may have advantages in this regard because of its ability to quantify both calcium and fat. The biggest disadvantage of DECT, compared to MRI, is the presence of ionizing radiation. The radiation dose is now significantly reduced with the development of several generations of DECT technology [31]. In this study, the radiation dose received by each patient for DECT scanning was about 4.6 mSv, which was a lower dose level, equivalent to twice the annual background radiation dose. Also, opportunistic examinations were possible because OP evaluations were performed at the same time as the lumbar spine scans, which reduced the radiation dose specifically for bone densitometry. Coupled with its advantages of short scanning time, multiparameter and multimodal imaging [32], DECT will have unique potential for the quantitative evaluation of OP in the future.

There were still some deficiencies in this study. First, the sample size was not large enough; Secondly, due to software reasons, we did not guarantee that the ROI shapes of the two measurements were consistent, which might cause some potential errors.

In conclusion, DECT can accurately quantify the BMAT of vertebrae, and has the same efficiency as MRI in the study of OP, which may be used as a supplementary method for fat quantification and OP evaluation.

## Data Availability

Data sets during the study period can be obtained from the corresponding authors upon reasonable request.

## Abbreviations

AUC: Area under the curve
BMAT: Bone marrow adipose tissue
BMD: Bone mineral density
DECT: Dual-energy computed tomography
DECT-FF: Fat fraction based on dual-energy computed tomography
FF: Fat fraction
ICC: Intraclass correlation coefficient
IDEAL-IQ: Iterative decomposition of water and fat with echo asymmetry and least-squares estimation
MRI: Magnetic resonance imaging
MRI-FF: Fat fraction based on Magnetic resonance imaging
OP: Osteoporosis
QCT: Quantitative computed tomography
ROC: Receiver operating characteristic
